# Quality of life in post-menopausal osteoporosis

**DOI:** 10.1186/1477-7525-3-78

**Published:** 2005-12-01

**Authors:** Maria Luisa Bianchi, Maria Rosa Orsini, Silvia Saraifoger, Sergio Ortolani, Giovanni Radaelli, Simonetta Betti

**Affiliations:** 1 Bone Metabolic Unit, Istituto Auxologico Italiano, IRCSS, Milan, Italy; 2 Institute of Psychology, Medical Faculty, University of Milano, Milan, Italy; 3 Unit of Medical Statistics, San Paolo Hospital, University of Milan, Italy

## Abstract

**Background:**

To evaluate the impact of osteoporosis on the patients' quality of life, particularly in the absence of fractures.

**Methods:**

100 post-menopausal women (age 50-85) - 62 with uncomplicated  primary osteoporosis and 38 with primary osteoporosis complicated by  vertebral fractures; all already treated - were studied using two validated  questionnaires: Qualeffo-41 for quality of life in osteoporosis, and Zung  for depression. Data were compared to those of 35 controls of comparable  age, affected by a different chronic disease (hypothyroidism).

**Results:**

Family history of osteoporosis and T-score of spine were similar  in the two subgroups of osteoporotic women. Body mass index, age at  menopause and education level were similar in the two subgroups of  osteoporotic women and in the control group.

The patients affected by osteoporosis perceived it as a disease affecting their personal life with undesirable consequences: chronic pain (66% of women with fractures and 40% of women without fractures), impaired physical ability, reduced social activity, poor well-being (21% of women without fractures) and depressed mood (42% of women irrespective of fractures). Overall, 41% of the women showed a reduced quality of life. On the contrary, in the control group only 11% reported a reduced quality of life.

**Conclusion:**

The quality of life of osteoporotic patients should be investigated even before fractures, in order to develop appropriate counselling, support and care interventions to help patients develop efficient strategies for accepting the disease and coping with it.

## Background

Osteoporosis is a very common disease of bone, and fragility fractures (i.e. fractures in the absence of relevant trauma) are its typical complication and the most common presenting sign. For many years, the diagnosis of osteoporosis was made only after the sudden occurrence of a fragility fracture.

The most frequent sites of bone fragility fractures are wrist, vertebrae, hip, ribs and humerus. While hip fractures have always been considered a cause of severe disability and loss of independence [1,2], there is now increasing awareness that all fractures substantially affect the patient's quality of life [3-5].

With the availability of new techniques of bone densitometry, and in particular of dual X-ray absorptiometry (DXA), osteoporosis was defined as the loss of bone mineral beyond a certain threshold, even in the absence of fractures. In 1994, the World Health Organization (WHO) defined it as "*a systemic skeletal disease characterized by a low bone mass and bone architectural derangements, leading to an increased fracture risk" *[6], and set the threshold of bone loss for osteoporosis, at least for post-menopausal women, at a T-score value of -2.5, as measured by DXA.

The aim of this study was to evaluate the impact of osteoporosis per se – independently of fractures – on the patients' quality of life; more specifically, to determine whether, in normal clinical practice, the awareness of being affected by osteoporosis has a special impact on the patients' perceived quality of life.

Indeed, most studies have been focused on the impact of fragility fractures. Our hypothesis, based on a very long clinical experience, is that the sheer awareness of a chronic, essentially progressive disease, with the well-known risk of bone fractures in one's future, has a negative effect on the subjective perception of the quality of life.

We thus involved a small control group of women of comparable age, affected by a different chronic disease (hypothyroidism), to determine whether osteoporosis could be considered to have a special impact on a patient's perceived quality of life.

## Methods

### Patients

One hundred post-menopausal women, aged 66 ± 8.7 years (age range 50–85), affected by osteoporosis with or without fractures, were enrolled consecutively at the outpatient clinic of the Istituto Auxologico Italiano over a period of about 4 months.

The diagnosis of osteoporosis was made on the basis of a reduction in bone mineral density (BMD) at spine and hip scan according to the WHO criteria, after a clinical and biochemical exclusion of other causes of bone loss. Only cases of primary osteoporosis were recruited. Secondary osteoporoses were excluded in order to avoid the interference of the primitive disease on the patient's quality of life. In all cases, osteoporosis was diagnosed before the present study, and all patients were receiving a specific treatment. 13 of the patients (13%) (9 in group A, without fractures; 4 in group B, with fractures) had used hormonal replacement therapy (HRT), starting at menopause and continuing for 3 to 6 years. No patients were on HRT at the time of the study. All the patients had had regular evaluation of 25-OH vitamin D serum levels also before the study, and they took vitamin D supplements if needed. 25-OH vitamin D levels were steadily normal (over 30 ng/ml) in all patients before and during the study. All the patients were living at home alone or with relatives, did not require personal assistance and were independent enough to come to the hospital's outpatient clinic.

The inclusion criteria included being able to autonomously read, understand and answer the simple questions asked in the questionnaires used in the study.

Beyond secondary osteoporosis, the exclusion criteria were the presence of other diseases affecting quality of life (e.g. cancer, moderate to severe chronic renal insufficiency, chronic respiratory diseases, cardiovascular diseases including uncontrolled hypertension, diabetes) and the presence of severe cognitive, visual or hearing impairments.

All the fractures were clinically symptomatic, and due to bone fragility and not to major trauma. For patients with a history of fractures, the last fracture must have occurred at least 6 months before the study, in order to evaluate only the impact of an established condition and not that of an acute phase.

### Control group

A control group of 35 women, affected by a different chronic disease (hypothyroidism, stable for at least 3 years) selected among the outpatients of the same Institute, was also involved and filled out the questionnaires. They were in the same age range of the patients, but without osteoporosis (mean BMD T-score -1.3 ± .3), back pain or fractures. Also these patients were aware of their disease, and were under treatment (levothyroxine replacement therapy). The inclusion and exclusion criteria were the same as for osteoporotic patients, apart bone loss.

### Methods

Bone mineral density was measured by DXA (Hologic QDR 2000) at lumbar spine and proximal hip. All vertebral fractures were confirmed by X-rays. All women underwent a lateral X-ray exam of the dorsal and lumbar spine at study entry: vertebral deformity was defined according to Genant's criteria [7].

The perceived quality of life was assessed by two standardized, internationally and nationally validated questionnaires: Qualeffo-41 for quality of life in osteoporosis, and Zung for depression.

Qualeffo-41 has five domains: pain, physical function, social function, general health perception, mental function (mood) [8,9].

The Zung Depression Scale is a self-report scale consisting of 20 items, each with a four-point severity rating [10]. The scale has often been used in the assessment of mood in people of mixed ages, and it has been validated in many studies [11-13]. When a score of 50 is used as the cut-off point, it is sensitive in detecting depression in epidemiological studies of persons over fifty years of age.

The patients and the controls were given the questionnaires during a routine follow-up evaluation, and filled them out on the premises at the Istituto Auxologico Italiano. All the patients had an interview with a psychologist, to exclude the presence of affective disorders that could influence the results of the tests.

The study protocol was approved by the Ethical Committee of the Istituto Auxologico Italiano.

### Statistics

All the questionnaires were answered completely and were analyzed according to the published scoring algorithm. Statistical analysis was performed using the SPSS statistical package 11.0 for Windows (SPSS Inc., Chicago, IL, USA). Data are expressed as mean ± SD or percentage. Student's *t *test for unpaired data, or the non parametric Mann-Whitney test and the chi-square, or the Fisher's exact test, as appropriate, were used to compare data between the two osteoporosis groups (with or without fractures) and the control group. Anova was also used to compare data among groups. Significance of multiple comparisons were adjusted by the Bonferroni's correction. Association between BMD, expressed as absolute value or T-score, and questionnaires' scores were evaluated by Pearson correlation coefficient in the patients affected by osteoporosis. Correlations were further adjusted for age and social status. A p value < 0.05 was considered to indicate statistical significance (two-tailed tests).

No significant differences were observed between the women who had used HRT (13 out of 100) and those who did not.

## Results

The main characteristics of the patients and the controls are summarized in Table 1. The patients were divided in two groups: *Group A: *62 women with uncomplicated osteoporosis, defined as a T-score <-2.5 according to the WHO criteria; *Group B: *38 women with osteoporosis, defined as above, but complicated by vertebral fractures. All 38 women had had at least 1 vertebral fracture, but none had had hip or other peripheral fractures.

**Table 1:** Main characteristics of patients and controls

	**controls**	**OP without fractures**	**OP with fractures**
number of women	35	62	38
age (years)	58.9 ± 7.9	64.5 ± 8.4	70.3 ± 7.8 *
BMI (kg/m^2^)	27.6 ± 3.4	25.6 ± 3.1	25.1 ± 2.9
age at menopause (years)	51.4 ± 2.9	51.9 ± 3.4	52 ± 3.8
			
*BMD T-score*			
lumbar spine	-1.3 ± .3 §	-3.2 ± .8	-3.3 ± 1
			
Femur	-1.1 ± .5 §	-2.6 ± .8	-3.1 ± 1.2°
*education (years of school)*			
<= 8	31% (11 women)	34.4% (21 women)	35.9% (14 women)
9–13	54.3% (19 women)	49.2% (30 women)	51.3% (20 women)
>13	14.2% (5 women)	16.4% (10 women)	12.8% (5 women)
			
Family history of OP	8.5% (3 women)ˆ	27.8% (17 women)	28.2% (11 women)

Data are expressed as mean ± SD and as percentages.

Student's *t *test for unpaired data or chi-square of Fischer's exact test.

OP = osteoporosis

* p < 0.05 versus controls and versus OP without fractures

° p < 0.05 versus OP without fractures

§ p < 0.02 versus OP without fractures and versus OP with fractures

ˆ p < 0.03 versus OP without fractures and versus OP with fractures

The control group and the two groups of women affected by osteoporosis were not significantly different. Body mass index (BMI), age at menopause, education level (evaluated as the number of school years), family history of osteoporosis were considered (Table 1). Of course, bone density in the control group was in the normal range. The T-score of spine was similar in the two groups of women with osteoporosis. The only differences were the mean age and the T-score values at hip, which were respectively a little higher and a little lower in the group with fractures (Table 1)

The evaluation of the Zung questionnaire revealed that some of the women affected by osteoporosis suffered symptoms of depression (40% of the women), but nobody reached the score of clinical depression from a psycho-pathological point of view. In the control group, a lower number of women had symptoms of depression (23% of the women) (Table 2).

**Table 2:** Scores of Zung test

**Controls**	**OP without fractures**	**OP with vertebral fractures**	**p-value**			
			ANOVA	OP w/out Fx vs. OP w/ Fx*	OP w/out Fx vs. controls*	OP w/ Fx vs. controls*
39.9 ± 10	48.6 ± 9	52.3 ± 12	<0.051	NS	NS	<0.05

**correlation between BMD T-score and Zung**				Coefficient -0.18		p-value = NS

Data are expressed as mean ± SD

*OP w/out Fx *= osteoporosis without fractures

*OP w/ Fx *= osteoporosis with vertebral fractures

* Mann-Whitney test

No significant correlation was found between the Zung questionnaire score and the BMD value, expressed as either the absolute value or the T-score (Table 2). 42% of women with osteoporosis had symptoms of depression according to the "mental function" domain of Qualeffo-41 [8,9], a result in accordance with the Zung test.

In the osteoporotic patients considered globally, the correlation between the BMD T-score value and the Qualeffo-41 score, adjusted for age, social status (e.g. education, marriage, living alone or not, etc.) and lifestyle habits (e.g. smoking, drinking), was significant (p < 0.001), independently from the presence of fractures. Three domains were particularly significant: physical function (p < 0.01), social function (p < 0.001), general health perception (p < 0.01) (Table 3).

**Table 3:** Correlation between BMD T-score and domains of Qualeffo-41 in 100 osteoporotic women

	**QUALEFFO-41 domain**					
	**pain**	**physical function**	**social function**	**health perception**	**mental function**	**global score**
**Crude correlation**						
Coefficient	-0.08	-0.32	-0.35	-0.27	-0.13	-0.29
p-value	NS	<0.01	<0.001	<0.01	NS	<0.01
						
**Correlation adjusted for age and social status**						
Coefficient	-0.12	-0.24	-0.32	-0.23	-0.12	-0.26
p-value	NS	<0.02	<0.001	<0.01	NS	<0.01

Pain was present in 50% of cases, independently from age, and in 26% for more than 10 hours a day. 25 women out of 38 with fractures (66%) and 25 out of 62 without fractures (40%) reported pain. The presence of vertebral fractures increased the score of the "pain" domain (Table 4). The Qualeffo score of pain was significantly higher in both groups of patients with osteoporosis with respect to the control group. In this latter group pain was present only in 2 cases (5,7%).

**Table 4:** Scores in five Qualeffo-41 domains

**Qualeffo-41 domain**	**Controls**	**OP w/out fractures**	**OP w/ fractures**	**p-value**			
				ANOVA	OP w/out Fx vs OP w/ Fx *	OP w/out Fx vs controls*	OP w/ Fx vs controls*
***Pain***	18 ± 4.5	40.7 ± 18.5	47.4 ± 20.5	<0.001	NS	<0.005	<0.005
***Physical function***	16.9 ± 3.4	21.6 ± 16.3	40.5 ± 20.6	<0.001	<0.0001	NS	<0.001
***Social function***	26.2 ± 9.3	36.2 ± 24.6	52.6 ± 27.3	<0.05	<0.005	NS	<0.002
***Health perception***	34.4 ± 12	58.5 ± 19.6	70.9 ± 22.1	<0.05	<0.004	<0.05	<0.0001
***Mental function***	35.9± 10.2	40.7 ± 18.5	47.4 ± 20.5	NS	NS	NS	NS
***Global score***	25.6 ± 8.9	39.5 ± 14.5	51.7 ± 18.7	<0.02	<0.001	<0.05	<0.001

Data are expressed as mean ± SD

*OP w/out Fx *= osteoporosis without fractures

*OP w/ Fx *= osteoporosis with vertebral fractures

* Mann-Whitney test

In the domain of physical function, 46% of the women under 65 years of age indicated the perception of a significant physical change, as did 65% of those over 65. The presence of fractures increased the perception of physical change (Table 4). The comparison with the control group revealed a slight difference with Group A, and a significant difference with Group B (Table 4).

In the domain of general health perception, 58% of the women had a sense of poor well-being. 13 women without fractures (21%) out of 62 reported a reduction of their health perception (Table 4). Comparing their present level of well-being with that of 10 years before, 58% of the women aged less than 65 indicated a deterioration, as did 83% of those aged 65 or more. These percentages were not significantly changed by the presence of fractures. In the control group, only 3 women (8,6%), aged 65 to 68 years, reported a reduction of their health perception (Table 4).

Overall, 41% of the women affected by osteoporosis had a reduced quality of life: 32% (23 cases) of the women with uncomplicated osteoporosis and 55% (21 cases) of the women with osteoporosis complicated by fractures. On the contrary, a reduction of quality of life was present in only 11,4% of the controls.

## Discussion

In an editorial published twelve years ago, Kanis et al already recognized the need "to rethink vertebral osteoporosis and to focus more closely than hitherto on the quality of life of these patients" [14]. However, only in these last years some information on the quality of life of osteoporotic patients has been gathered, generally after fractures [15-19].

Most information has been collected thanks to the efforts of some researchers to develop specific instruments to test the physical and emotional disability generated by the disease. Generic instruments available for measuring quality of life (such as SF-36) are useful to evaluate health in general but they lack disease specificity [20-22]. More recently, some specific instruments were developed to give a more accurate measure of the quality of life in osteoporosis. One of the first was Qualeffo-41, which has been translated and validated in different languages including Italian [8,9]. This questionnaire has proven to be repeatable, coherent, and able to discriminate between patients and controls. In the last years other specific questionnaires have been developed, but not all were as extensively used and validated in different countries as Qualeffo-41 [23-26]. This is the reason why we decided to use the Qualeffo-41 questionnaire.

While most previous studies included only women with osteoporosis complicated by fractures, we chose to include also a group of patients with a diagnosis of osteoporosis but without fractures, a condition which is usually considered asymptomatic. And we included also a control group of women of comparable age, affected by a different chronic disease of comparable severity and essentially asymptomatic (hypothyroidism), who were also on a long term therapy and in a stable clinical condition.

In previous studies, it was demonstrated that vertebral fractures are associated with reduced quality of life and that physical function and emotional status are negatively affected [27,28]. The use of specific questionnaires showed that the reduction of quality of life depends on the number of vertebral fractures and on their location within the spine, with significantly different scores [28,29].

In our study we did not try to evaluate this particular aspect, given the relatively small sample, but we considered the vertebral fractures as a whole, without considering the number of vertebral fractures and their location.

To the specific test measuring the perceived quality of life (Qualeffo) in osteoporosis, we also added the Zung Depression Scale to measure depression. An essential prerequisite for the correct interpretation of the results is to be able to discriminate between a condition of reactive depression, such as is frequently encountered in chronically ill subjects, and that of endogenous depression (melancholia). Both the Zung questionnaire and the related domain of Qualeffo-41 showed that a depressed mood was present in about 40% of our patients, independently of fractures, while nobody had a major depressive disorder as defined by the DSM-IV [30], considering both their past and their recent history.

In accordance with the study of Oleksik et al. [29], the mental function of our patients was not influenced by the presence of the disease, whether they had suffered fractures or not. This is an important aspect, as it strongly supports the hypothesis that the physical constraints of the disease and not the mental impairment cause the reduction in physical abilities, the consequent reduction in social activities and in autonomy, the permanent modification of body image, and the perception of general health. Moreover, the comparison with a control group of women affected by another chronic disease confirmed the lack of mental function involvement.

Another extremely important aspect evidenced by our study was the presence of pain in a significant number of patients before the occurrence of vertebral fractures (clinical or even morphometric). Osteoporosis is generally considered a silent disease before the occurrence of fractures, but we found that pain was often present in the group of women without known fractures (a clear difference with respect to the control group). Chronic pain in osteoporosis is poorly considered even in the presence of vertebral fractures [31-33], and it is obviously even more underestimated without them. There is now ample consensus that undertreated chronic pain may become "a disease within the disease" and is often a cause of subclinical or clinical depression [34,35]. Our study demonstrates that physicians dealing with osteoporotic patients must pay serious attention to the problem of chronic pain, and even in the absence of fractures.

Recently, Dhillon et al. demonstrated that women with osteoporosis have a reduced health-related quality of life, independently of prior fractures [36]. In our study, the use of a test (Qualeffo), specifically designed for osteoporosis demonstrated even better the effect of the disease itself, without the presence of its main complication (fragility fractures). We think that our results are even more relevant because of the comparison with a control group affected by a different chronic disease.

A reduced quality of life was present in our patients notwithstanding their stable, non-alarming condition, with the diagnosis of osteoporosis back in the past. All of them were following a standard therapy for osteoporosis, well known to be effective in the majority of cases. The presence of a therapy is considered a factor which influences positively a patient's quality of life [37,38], but notwithstanding this, in our patients, the therapy for osteoporosis was not able to completely eliminate the impact of the disease on the perceived quality of life.

## Conclusion

Any chronic disease can induce a negative perception of one's quality of life, especially considering the future and the risk of losing independence or suffering pain. We aimed at determining whether osteoporosis, a very common disease, could have a special impact on a patient's perceived quality of life.

The results of the Qualeffo and Zung tests revealed significant differences between the osteoporosis patients and the control group. Patients with osteoporosis, even in the absence of fractures, had a more depressed mood and a lower quality of life.

Osteoporosis was perceived by our patients as a disease leading to severe discomfort and/or disability, and affecting different aspects of personal life with a variety of undesirable consequences, such as chronic pain, reduced physical ability, reduced social activity, poor well-being, and depressed mood. The fear of losing autonomy and independence was extremely high.

On the basis of our results in a relatively small sample (100 patients), we think that the quality of life of osteoporotic patients should be thoroughly investigated even before the occurrence of fractures, in order to develop the appropriate intervention (e.g. counseling, support and care) in all the different phases of the disease. This will help patients to develop more efficient strategies for accepting the disease and coping with it. The information obtained through the use of appropriate questionnaires could be a powerful instrument for the physician or caregiver in the global management of osteoporosis.

## Authors' contributions

MLB designed the study, contributed to select the patients, and actually wrote the article. MRO, SB made the psychological interviews and evaluation and calculated the test scores. SS administered the questionnaires, performed the densitometric (DXA) analysis, and prepared the database. SO contributed to select the patients with osteoporosis. GR performed the statistical analysis. All authors have read and approved the final version of this paper.
